# Conventional and Nonconventional Sources of Exosomes–Isolation Methods and Influence on Their Downstream Biomedical Application

**DOI:** 10.3389/fmolb.2022.846650

**Published:** 2022-05-02

**Authors:** Olga Janouskova, Regina Herma, Alena Semeradtova, David Poustka, Michaela Liegertova, Hana Auer Malinska, Jan Maly

**Affiliations:** Centre of Nanomaterials and Biotechnology, Faculty of Science, Jan Evangelista University in Ústí Nad Labem, Ústí Nad Labem, Czech Republic

**Keywords:** exosomes, isolation methods, sources of exosomes, storage of exosomes, biomedical application of exosomes

## Abstract

Despite extensive study of extracellular vesicles (EVs), specifically exosomes (EXs) as biomarkers, important modulators of physiological or pathological processes, or therapeutic agents, relatively little is known about nonconventional sources of EXs, such as invertebrate or plant EXs, and their uses. Likewise, there is no clear information on the overview of storage conditions and currently used isolation methods, including new ones, such as microfluidics, which fundamentally affect the characterization of EXs and their other biomedical applications. The purpose of this review is to briefly summarize conventional and nonconventional sources of EXs, storage conditions and typical isolation methods, widely used kits and new “smart” technologies with emphasis on the influence of isolation techniques on EX content, protein detection, RNA, mRNA and others. At the same time, attention is paid to a brief overview of the direction of biomedical application of EXs, especially in diagnostics, therapy, senescence and aging and, with regard to the current situation, in issues related to Covid-19.

## Introduction

Exosomes (EXs) are nano-sized extracellular vesicles (EVs) released from a subdivision of previous endosomes. EXs were first reported in the mid-1980s (Pan and Johnstone, 1983) (Pan and Johnstone, 1983). Initially, researchers assumed that EXs simply served as “garbage bags” to enable cells to get rid of unwanted components (Lin et al., 2015). However, increasing evidence has proven that EXs play an important role in many cellular processes, and provide a unique mode of cell-to-cell communication and also influence both physiological and pathological processes (Yamashita et al., 2016; Ailuno et al., 2020). EXs are released from many cell sources.

They can also be found in most body fluids (Théry et al., 2002). In recent decades, an increasing number of studies have revealed many other sources of EXs, e.g., nonconventional - animal product derived EXs, bacterial, fungal, parasitic, plant derived EXs (Schuh et al., 2019). In general, due to their biological function, EXs play an increasingly important role in diagnosis, aging studies, and as various therapeutic agents (Aheget et al., 2020).

In this review we focus mainly on a brief summary of various sources (conventional and nonconventional) of EXs, typical isolation methods, widely used kits and new “smart” technologies with emphasis on the influence of isolation techniques on EX content, protein detection, RNA, mRNA and others. At the same time, attention is paid to a brief overview of the directions of biomedical application of EXs, especially in diagnostics, therapy, senescence and aging and, with regard to the current situation, in issues related to Covid-19.

## Exosomes–Biogenesis, Composition, Function and Sources

EXs as nano-sized extracellular vesicles have a diameter of about 30–150 nm (Tanziela et al., 2020) and density of 1.13–1.19 g/ml (Deb et al., 2021), dependent on their place of origin, as well as a lipid bilayer structure in the cell (Kalluri and LeBleu, 2020). EXs were first described in maturing mammalian reticulocytes (Pan and Johnstone, 1983). They release transferrin receptors (TFR) throughout small vesicles into the extracellular environment (Lin et al., 2015; Batista and Melo, 2019). Ever since Valadi et al. first stated in 2007, that EXs carry RNAs, the composition and function of these vesicles have been extensively studied (Valadi et al., 2007).

Now it is known that EXs contain various types of molecular constituents, such as nucleic acids, proteins, lipids, cytokines, transcription factors, regulatory RNAs and some other bioactive substances (Jing et al., 2018; Jeppesen et al., 2019; Zhang et al., 2020). Structurally, EX surfaces are rich in transmembrane proteins, receptors, and other functional molecules (Jafari et al., 2020). EXs have a membrane structure with a lipid bilayer in which lipids such as polyglycerol, phospholipid, ceramide, sphingomyelin, cholesterol and phosphatidylinositol are included.

The biogenesis of EXs starts with endocytosis of cell membranes, including receptor-mediated endocytosis, nuclear endocytosis of inclusion proteins and pinocytosis (Kim et al., 2021). The early endosomes are formed by the endocytosis and later matured into late endosomes. EXs carry information from the cells of origin thanks to continuous intracellular exchange of substances by invagination. The invagination in the late endosomes leads to the formation of intraluminal vesicles (ILVs), so called future EXs (Kalluri and LeBleu, 2020). ILVs are finally secreted as EXs through multivesicular bodies (MVBs) fusion to the plasma membrane and exocytosis. The exocytosis permits EXs to be released with their characteristic lipid bilayer structure (Dai et al., 2020; Jiang et al., 2020). The whole process of EX biogenesis is strictly regulated (Aheget et al., 2020).

The main elements that influence the biogenesis of EXs include certain biological factors that are essential for their formation, secretion, and yield. Studies have suggested that EX yield depends for example on the cell type, biotic and abiotic stress or that confluence of cell cultures also plays a critical role (Gurunathan et al., 2019; Aheget et al., 2020). As in all membrane vesicular processes, lipids are also important players in the biogenesis of EXs (Pegtel and Gould, 2019).

EXs are released from many cell sources, such as lymphocytes, dendritic cells, epithelial cells, endothelial cells, mast cells or neurons. They can also be found in most body fluids inclusive of blood, saliva, urine, breast milk, amniotic fluids, hydro thoracic fluids, ascitic fluid and in the culture medium of almost all cell types (Théry et al., 2002). These “conventional” sources undoubtedly provide an important basis for understanding the roles of EXs in a number of physiological processes, as well as for their subsequent possible use in biomedicine (Jing et al., 2018).

**FIGURE 1:**
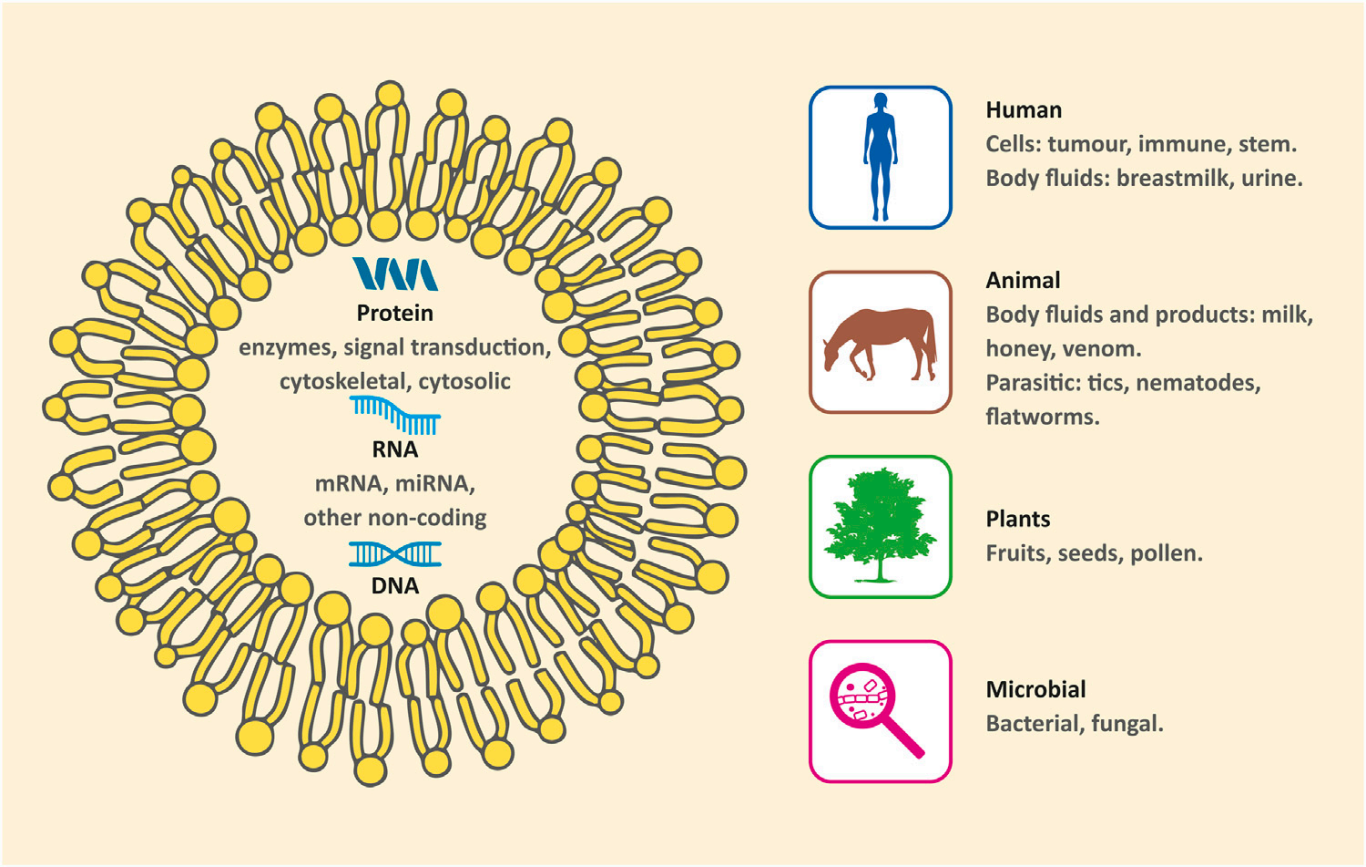
Sources of exosomes.

However, in the last decades, an increasing number of studies have revealed that many other sources exhibit remarkable characteristics and clarify some biological processes. Moreover, they could also be useful alternatives for medical applications. Thus, EXs can be categorized by sources as conventional (human/mammal) and nonconventional (nonhuman/nonmammal). The latter can be further subdivided into 1) animal product derived EXs, 2) bacterial, fungal, and parasitic EXs, and 3) plant derived EXs (Figure 1) (Schuh et al., 2019).

It is generally accepted that EXs play a crucial role in many physiological and pathological processes (Yamashita et al., 2016; Ailuno et al., 2020). A considerable part of the literature indicates that EXs display a wide range of functions, which depend on their cell or tissue of origin. Especially, EXs from certain types of immune cells may ensure adaptive immune responses to pathogens or tumors (Schwarzenbach and Gahan, 2021). Moreover, EXs are usually able to guard their loads against removal from the body or destruction, due to their dual layered membrane and nano-size (Tanziela et al., 2020). Therefore, they have the potential to be used to deliver therapeutic molecules (e.g., small interfering RNA, microRNAs) or small molecule drugs (e.g. doxorubicin, curcumin) to target cells for treating various types of disease. Exosomal chemical or biological modification can also increase the characteristic therapeutic competence of EXs (Tian et al., 2014).

## Nonconventional Sources of EXs

Most of the exosome production and isolation techniques focus on the use of mammalian sources (cells, tissues, and body fluids). These conventional sources provided a key understanding of the role of EXs in basic (mammalian) physiological processes, and their potential role in biomedicine as therapeutic agents. Extracellular vesicles are highly evolutionary conserved across all eukaryotic organisms (Gill et al., 2019). Non-mammalian resources of EXs are gaining in popularity for their interesting characteristics and complex roles (ranging from inter-species interactions to inter-kingdom communication) providing possible alternatives for biomedical applications (Schatz and Vardi, 2018; Schuh et al., 2019).

EXs, representing a common mechanism for mediating communication in host-parasite interactions, are of particular relevance to biomedicine, since they are known to act as vectors for virulence factors and effector proteins from parasites to their hosts (Zhou et al., 2018; Nawaz et al., 2019; Wu et al., 2019; Xander et al., 2020). EXs were shown to mediate pathogenic processes through influencing gene expression and host response of the immune system (Nawaz et al., 2019). However, EXs are produced by the host cells in response to the parasite, thus a bidirectional regulatory effect on parasitic infection is present. Isolation of EXs provides novel approaches to diagnostics and possible treatment of various parasitic infections (Wu et al., 2019).

EXs and exosome-like vesicles have recently been isolated from bee glandular secretion products (honey, royal jelly, and bee pollen) and their bacteriostatic, bactericidal, and biofilm-inhibiting effects were demonstrated together with the ability to increase migration of human mesenchymal stem cells (MSCs) *in vitro* (inter-kingdom activity) (Schuh et al., 2019).

EXs have been isolated from lyophilized snake venom (Carregari et al., 2018) and analyzed by mass spectrometry-based proteomic approach, suggesting their role in the cytotoxicity of the venom, and providing new insight into the envenomation process (Carregari et al., 2018).

From studies on model organisms, *D. melanogaster* and *C. Elegans* (Beer and Wehman, 2017; Russell et al., 2020), it seems that extracellular vesicles (EVs) and EXs play a conserved role in developmental processes (i.e., carrying patterning morphogens) and presumably in adult mating-specific behavior. Moreover, the functional role of extracellular vesicular transport of Wnt proteins in *D. melanogaster* and similarly in murine model was demonstrated (Gross et al., 2012).

Other numerous non-mammalian eukaryotic organisms have been shown to use EXs/EXs-like vesicles; lower vertebrates, i.e. danio (Verweij et al., 2019), *xenopus* (Danilchik and Tumarkin, 2017), turtles (Zhu et al., 2019), molluscs, i.e. oysters, where EXs have been found to be involved in reparation of the outer shell (Johnstone et al., 2015), protozoa (Lin et al., 2019) and fungi (Rizzo et al., 2020). However, many possibly interesting sources of EXs with diagnostic or clinical potential remain to be discovered.

Nowadays, in addition to animal sources of EXs, plant-derived EXs come into focus. The term “EXs” has traditionally been used for animal vesicles. Similar structures have recently been discovered in plants as well, they are called plant exosome like vesicles (PELVs) and they seem to possess similar size (50–150 nm), structure and analogical function, but research is still at the beginning. Since fruits, seeds, leaves and roots of various plants are a natural part of human nutrition, PELVs, included in plant tissues, are considered to have low toxicity and immunogenicity. For research purposes, iPELVs can be isolated in large quantities at very low cost from juiced plant parts or apoplastic fluid, avoiding lengthy isolation protocols.

As an example, traditional plant model organisms, such as *Arabidopsis thaliana* or Nicotiana tabacum, have been used for PELVs characterization (Rutter and Innes, 2017). Research of plant vesicles is very specific, due to its potential as nanoplatforms for drug delivery (Dad et al., 2021).

Recently, PELVs have been widely studied for their therapeutic potential. For example, PELVs isolated from ginger protected mice from alcohol-induced liver damage (Zhuang et al., 2015). Ginger (Zhang et al., 2016) grape (Ju et al., 2013) and broccoli (Deng et al., 2017) PELVs enhanced intestinal repair and reduced acute colitis in murine models. Moreover, ginger PELVs mediated transport of therapeutic agent Doxorubicin, enhanced chemotherapeutic action and inhibited growth of tumors better than free drug (Zhang et al., 2016).

Significant anticancer potential of Dendropanax morbifera PELVs was also observed. Vesicles isolated from this Korean shrub reduced growth of mouse melanoma (Lee et al., 2020). Inhibition of cancer cell line proliferation of PELVs isolated from fruits like grapefruit or lemon was observed *in vitro* using various tumor cell lines (Raimondo et al., 2015). Lemon PELVs inhibit cancer cell proliferation in different tumor lines, but, *in vivo*, promotion of apoptosis in the fight against chronic myeloid leukemia has been proved (Raimondo et al., 2015). Moreover, miRNAs from grapefruit and ginger PELVs were reported to promote the growth of *Lactobacillus* rhamnosus in mice gut, boosting the immune response and wellbeing of the mice gut (Mu et al., 2014).

The regeneration and wound healing potential of PELVs has also been investigated. In vitro studies, using wheat-derived PELVs, the expression of collagen mRNA was detected as well as increased vascular formation, providing new possibilities for skin regeneration strategies (Şahin et al., 2019).

Use of plant derived nanovesicles is still at the beginning, concealing a large future potential for broader use. The latest trend in this field is the production of transgenic plant-derived virus-like agents for the production of vaccines (Chen and Lai, 2013), e.g. plant-derived virus-like vaccine against influenza is now an ongoing Phase III clinical trial (Pillet et al., 2019).

## Exosome Isolation Methods

EXs isolation techniques are based on three basic biophysical principles; 1) Size and density; 2) Polymer precipitation; 3) Immunoaffinity capture (Supplementary Table S1). Comparisons of recent strategies and technologies for EXs isolation and purification are described in many reviews (Tang et al., 2017; Chen et al., 2019; Sidhom et al., 2020; Yang et al., 2020). Recently Chen et al. focused on comparison of isolation methods with emphasis on the EXs yield and purity (Chen et al., 2022). The very specific “smart” isolation method, which combines the aforementioned principles, are microfluidics-based methods(Yu et al., 2018; Yang et al., 2020; Zhou et al., 2020). Currently, there are many EXs isolation kits available on the market. Many studies comparing isolation methods were performed, where characteristics like exosome yield, size distribution, marker-based assessment and RNA or protein quantitation and qualification were taken under the scope (Patel et al., 2019). All isolation methods are still under progress with the goal to establish a versatile, reliable and robust isolation method that is a crucial aspect for further EXs utilization in non-invasive diagnostic and therapeutic approaches (Patel et al., 2019; Deb et al., 2021) target drug delivery systems or novel modulators for vaccination (Deb et al., 2021; Kim et al., 2021; Song et al., 2021).

### Size and Density-Based Isolation Methods

In general, EXs isolation is based on removing larger constituents like cells, dead cells and cell debris followed by elimination of smaller elements like macromolecules from the sample. This can be performed by 1) ultracentrifugation (UC); 2) density gradient centrifugation; 3) ultrafiltration; or 4) size-exclusion chromatography (SEC).

Ultracentrifugation is currently signed as the gold standard for EXs isolation (Zhang et al., 2020). (Chen et al., 2019)lower speed, EXs are obtained by centrifugation at min. 100,000xg for at least 1 h. Centrifugation time, centrifugal force or rotor type can affect the yield and purity of the EXs, but also their destruction and aggregation (Cvjetkovic et al., 2014; Livshts et al., 2015). The purest EXs are obtained by density gradient centrifugation. Sucrose and Iodixanol are two commonly used media to create the gradient, when Iodixanol is more stable and less viscous than sucrose, which results in shorter centrifugation times (Ford et al., 1994; Van Veldhoven et al., 1996). Moreover, iodixanol gradient enables separation of EXs from some types of retroviruses (Cantin et al., 2008). Advantages of centrifuge-based methods are their purity and suitability for large volume isolation. Disadvantages are high instrumental costs, time consuming procedures and non-negligible loss of the EXs during the procedure (Gurunathan et al., 2019; Zhou et al., 2020).

Ultrafiltration separates EXs from the sample by membranes with different molecular weight cut-offs. By simple adjusting of the order and filter size, ultrafiltration allows to sort specific subsets of EXs with defined particle sizes with yield comparable to that of the ultracentrifugation method (Heinemann and Vykoukal, 2017). Moreover, ultrafiltration-based exosome isolation dramatically shortens processing time with minimal equipment requirement.

Advanced methods of tangential stream filtration utilize so-called tangential flow streams. The feed stream flows parallel to the membrane face. The applied pressure causes a defined fraction of the flow stream to pass through the membrane filter according to the pore size. During the tangential flow filtration procedure, several membranes are included and simultaneously several fractions of the sample can be collected (Heinemann and Vykoukal, 2017).

Size-exclusion chromatography allows sequential elution of different-sized extracellular vesicles fractions from one column, where small particles remain trapped in the pores of the statical phase and bigger particles are eluted along the pores with the mobile phase (Zhang et al., 2020). This method is successful in the separation of EXs from proteins, but may doped with particles of similar size like aggregates, lipoparticles or particulate matter (Böing et al., 2014; Gurunathan et al., 2019). Based on this principle, there are two commercial EXs separation kits on the market, qEV (iZON) and PURE-EVs (Hansa Biomed).

### Polymer-Based Precipitation

The objective of those methods is to reduce the EXs solubility by introducing water soluble, slightly amphiphilic polymer molecules. Those molecules interact with water molecules surrounding the EXs to create a hydrophobic micro-environment, resulting in exosome precipitation. Obviously, the precipitation methods are based on 1) Polymer precipitation; or 2) Aqueous two phase systems separation (ATPSs).

Several commercially available kits are based on polymer precipitation: mExoPrep (Hansa BioMed), Total Exosome Isolation Reagent (TEI) (Invitrogen), ExoQuick (System Biosciences), Exosome Purification Kit (Norgen Biotek), miRCURY, PureExo (101Bio) and Exosome Isolation Kit (Exiqon).

In general, cells, dead cells, apoptotic bodies and debris are removed before EXs precipitation by filtration or centrifugation. Typical precipitation methods use polyethylenglycol, with molecular weights in range 6–20 kDa and overnight incubation at 4 °C. Finally, the EXs are harvested via low-speed centrifugation. Compared to ultracentrifugation methods, EXs precipitation is fast and relatively easy to provide (Zhang et al., 2020). However, water-excluding polymers can also precipitate other water-soluble materials such as nucleic acids, lipoproteins, proteins, and even viruses (Sim et al., 2012; Kimura et al., 2019). To overcome the problem with impurities, lectin agglutination can be performed. Lectins are a family of proteins that recognise carbohydrate moieties with very high specificity. Similar to PEG molecules, lectins bind to carbohydrates on the surface of the EXs, causing them to precipitate out of the solution. Therefore, only highly glycosylated impurities can be co-precipitated with EXs utilizing the lectin-induced precipitation (Samsonov et al., 2016; Doyle and Wang, 2019).

The aqueous two phase system for EXs separation (ATPSs) is a more advanced precipitation method yielding purer harvested EXs compared to basic polymer precipitation. ATPSs are based on separation of different kinds of particles in different phases. After mixing samples with dextran and PEG solutions and using low speed centrifugation, the dextran solution forms the lower phase with accumulated EXs. The upper phase, which is a relatively more hydrophobic solution of PEG, preferentially accumulates proteins and other macromolecular complexes (Shin et al., 2015; Yang et al., 2020).

### Immunoaffinity Capture

All aforementioned methods allow only the nonspecific isolation of the EXs. Immunoaffinity methods remain the only method to distinguish between subgroups of vesicles or fishing of preferred subgroups from the sample. Most of the targeted moieties are proteins tetraspanins CD9, CD81 and CD63, because those markers are expressed on nearly all EXs (Clayton et al., 2001). Other markers like Rab5, CD82, annexin TGS101, or Alix have been also published for selective exosome isolation (Andreu and Yáñez-Mó, 2014; Willms et al., 2018; Liu and Su, 2019). There are two principles of EXs isolation based on immunoaffinity capture: 1) Enzyme-linked immunosorbent assay (ELISA); 2) Immunoprecipitation.

Enzyme-linked immunosorbent assay (ELISA) requires antibodies against an antigen of interest immobilized on the microplate surface. Unbound elements are washed away while immobilized EXs are detected by another antibody containing a detection tag. When the quantitative standards are available, a calibration curve can be measured and therefore ELISA can also be utilized as a quantitative method (Doyle and Wang, 2019). Commercial ELISA kits are available for the most common antigens, CD9, CD81 and CD63, e.g., ExoELISA Complete Kit (System Biosciences), Human Exosome ELISA colorimetric Kit (Novus Biologicals), Overall Exosome ELISA Kit (Abnova), ExoSense ELISA Kit (EsiBio), ExoQuant (BioVision) and many others.

For immunoprecipitation, antibodies or recombinant proteins should be fixed to solid state matrices, where the submicron-sized magnetic or polymeric beads are the most common option. This method exhibits high yield efficiency and very good sensitivity, due to its large surface. Moreover, this method enables processing of large starting volumes of samples, EXs preconcentration and also their surface immunoanalysis (Li et al., 2017). Therefore, this method is suitable for future use in diagnostics through the detection of disease-specific marker. s.

Again, there are many commercial kits on the market, such as Exosome Isolation Kit (Miltenyi Biotec), Exosome Capture beads (Abcam), ExoCap Streptavidin Kit (JSR Life Science Company), Exosome-Human Isolation/Detection Reagent (Invitrogen) as well as Exo-Flow Capture Kit (System Biosciences) and others.

The non-antibody affinity beads represent a universal immunoprecipitation based option for EXs isolation. The technology, named EVtrap (extracellular vesicle total recovery and purification) is based on beads modified with a combination of hydrophilic and aromatic groups that exhibit high affinity toward the lipid bilayer of the EXs (Iliuk et al., 2021). So-called chemical affinity EVtrap beads (Tymora analytical operations) are already available on the market.

### Conclusion Remarkes to Conventional Isolation Methods

In conclusion, EXs isolation is not a completely solved process, and is still varying with respect to the source of the EXs, or the purpose for which they are isolated.

Human EXs, animal exosome-like vesicles or plant exosome like vesicles (PELVs) exhibit very similar properties on the interkingdom level. Therefore, many methods such as ultracentrifugation, ultrafiltration, size-exclusion chromatography, or precipitation are universal tools for EXs isolation.

Ultracentrifugation is a gold standard for EXs isolation and it is also universal for isolation from all known sources such as from plants (Urzì et al., 2021), animal secretions (Schuh et al., 2019), cell cultures (Théry et al., 2006) or human body fluids (Keller et al., 2011). The limit of this method is the high amount of exosomal source required. In the case that only a small volume of the exosomal source is available, precipitation based methods offer a good solution. This method is also universal for a wide spectrum of exosomal sources such as plants (Suharta et al., 2021), cell culture and body fluids. As there is a wide spectrum of commercial kits on the market, several comparative studies have been performed e.g., (Martins et al., 2018).

The most specific isolation method is based on immunoaffinity recognition. Commercial kits based on this princple are targeted to a specific marker (mostly human), therefore, cannot be applied on all EXs sources. Moreover, every immunoaffinity isolation is also optimized for the type of body fluid or the target of interest and the purpose of investigation. These procedures are mostly connected with cancer disease. Because EXs can play a very important role as the biomarkers in the cancer diagnosis and prognosis (Czystowska-Kuzmicz and Whiteside, 2021)a variety of studies have been already published e.g.(Whitesides, 2006; Hong et al., 2014).

The most advanced method for EXs isolation is microfluidics because this access for isolation also enables the EXs characterization in one microdevice (He et al., 2014).

### Microfluidics

A new and rapidly evolving approach to exosome isolation, as well as detection and analysis, is utilizing microfluidic technologies. Microfluidics in general refers to the study and manipulation of fluids that are geometrically constrained in channels with dimensions of tens of micrometers. At this scale, fluid behavior differs from conventional flow theory and the impact of volumetric forces is overtaken by the surface ones (Whitesides, 2006)Miniaturization, automation and precise fluidic control achieved by implementing microfluidics leads to many benefits such as reduced sample consumption, lower reagent volume, enhanced purity or the possibility to combine multiple processes into a single step and onto a single microfluidic chip. Having effective on-chip solutions for exosome related processes would minimize cost in the long run, speed-up otherwise time consuming isolations and enable point-of-care acquisition and diagnosis. These are all crucial steps in bringing EXs to everyday laboratory and clinical praxis (Liga et al., 2015; Contreras-Naranjo et al., 2017; Jia et al., 2019).

Different authors categorize numerous microfluidic chips for exosome isolation developed in recent years into various categories and subcategories (Chen et al., 2019; Wang et al., 2021). However, at the basic level, there are currently only two possible approaches. Immunoaffinity is the common denominator of an otherwise broad range of various clever microfluidic device constructions.

The first approach, microfluidics, utilizes antibodies and immune specificity of involved reactions (Liu et al., 2018; Chen et al., 2019) to isolate EXs.

The second approach aims at the physical properties of EXs, size, surface charge and density. These microfluidic chips oriented at EXs’ physical properties utilize standard processes such as filtering through membranes (Liang et al., 2017), sieving/trapping at nanostructures (Yasui et al., 2017), and using effects of electric field (Heineck et al., 2017; Ibsen et al., 2017). They also utilize more “niche principles” such as viscoelastic separation (Liu et al., 2017; Zhou et al., 2019), deterministic lateral displacement (Liu et al., 2017)or acting on the particles *via* surface acoustic waves (Ku et al., 2018; Wang et al., 2021).

Both described approaches have their pros and cons. Devices using immune-affinity can be very efficient and can achieve high throughput, but they are not yet suited for all exosome types, and usually require complicated sample pre-treatment. Antibody labeling may also induce impurities. On the other hand, devices aimed at physical properties are label-free and capable of processing complex bodily fluids, but their usage generally leads to higher percentages of contaminants.

The optimal solution may lie in combining the best of both approaches and using multiple isolation principles in a single chip. Moreover, the key potential of microfluidic devices is the possibility to integrate both exosome isolation and characterization onto the same chip, thus making a huge step in enabling POC acquisition and unlocking all the advantages described earlier.

For the purpose of this all-encompassing review, we have only briefly scratched the surface of both realized and potential benefits that microfluidics could bring to the field of exosome research. However, there are several comprehensive reviews from recent years that are focused solely on microfluidics and go into great detail in their description and comparison (Liga et al., 2015; Contreras-Naranjo et al., 2017; Jia et al., 2019; Choe et al., 2021; Ding et al., 2021; Wang et al., 2021).

## Storage

The following crucial topic following the EXs isolation is their storage. Their stability for extended storage is low. The technology of conservation of EXs, which protects their biological activities and makes them suitable for transport and subsequent clinical applications, is therefore a major challenge (Aheget et al., 2020). At present, applied protection techniques mostly include freezing, lyophilization and spray drying (Zhang et al., 2020).

Cryopreservation is a storage technique in which the temperature is reduced below the temperature necessary for biochemical reactions, to maintain the functional stability of biological particles. Typically, temperatures of 4°C, −80°C and −196°C are used (Zhang et al., 2020). Nevertheless, a major disadvantage of this method is the “susceptibility to frost injury”. The “frostbites” usually refer to the annexation of osmosis during the freezing process and to the formation of ice crystals inside the biological particles. To overcome this shortcoming, one or more antifreeze mixtures, so called cryoprotectants with appropriate concentrations, are frequently selectively added (Jeyaram and Jay, 2018; Kusuma et al., 2018; Bahr et al., 2020).

These cryoprotectants are usually divided into two main types, based on their permeability: 1) intracellular compounds (penetrating cryoprotectants) and 2) extracellular agents (non-penetrating cryoprotectants). The penetrating cryoprotectants (e.g., ethylene glycol and dimethyl sulfoxide) have a low molecular weight and can penetrate the membrane into the exosome and prevent the formation of ice crystals and subsequent membrane rupture (Bahr et al., 2020). Non-penetrating cryoprotectants, which do not penetrate the membrane (e.g., trehalose, sucrose and other carbohydrates), because of their high molecular mass, can form hydrogen bonds with water (Kusuma et al., 2018).

Many studies, based on obtained proofs, suggested combining both penetrating and non-penetrating cryoprotectants for higher effectivity (Ha et al., 2005; Bahr et al., 2020). It is necessary to adjust the concentrations of cryoprotectants, as it has been shown that excessively low concentrations can lead to freezing shock (the damage caused by the freezing process), while excessively high concentrations can be toxic (Best, 2015).

Freeze-drying, or lyophilization, is a method of pre-cooling materials that contain moisture. The lyophilized material freezes below the freezing point to a solid, directly sublimes the ice in vacuo and removes it as water vapor. This technology is divided into three main phases: 1) the pre-freeze phase; 2) the sublimation drying phase and 3) the analytical drying phase (Yang et al., 2020). The freeze-dried exosomal lyophilized powder can effectively reduce the complexity of storage conditions. Because the dehydration and drying of the products is completed under low temperature and vacuum conditions, the material can better retain its original activity and possible damage is significantly reduced. In addition, such a material can be easily maintained in a stable, storable state and can be easily reconstituted by simply adding water.

Similar to cryopreservation, the freeze drying and dehydration stress generated during this process can lead to destructive effects on the structure of EXs. For this reason, the use of “lyoprotectants” is proposed to protect EXs and their cargo (Nireesha et al., 2013). The most commonly used stabilizers (lyoprotectants) are disaccharides, such as lactose, sucrose and trehalose. Some studies have shown that trehalose appears to be the most effective disaccharide for preserving EXs during lyophilization, because it effectively prevents aggregation of EXs during lyophilization, increases their colloidal stability and does not alter their morphology (Bosch et al., 2016; Charoenviriyakul et al., 2018).

Spray drying is an approach that is systematically applied to the drying of various materials. After spraying the exosome solution in the drying room, the moisture evaporates rapidly on contact with hot air to give a dry powder. Factors that affect the stability of EXs during the process are mainly atomization pressure and outlet temperature. Compared to lyophilization, spray drying is faster and, since the drying method is designed to be continuous, it is also more economical (Kusuma et al., 2018).

The spray drying process is composed of five steps: 1) concentration, the material is normally concentrated before introduction into the spray dryer; 2) atomization, according to the required properties of the dried product, the atomization phase creates optimal conditions for evaporation; 3) droplet–air contact, in a special chamber, the atomized liquid then comes into contact with the hot substance of the gas, which causes evaporation of 95% of the contained water (in droplets) within a few seconds; 4) droplet drying, this takes place in two phases; during the first phase (it proceeds at a slightly constantspeed) there is enough moisture in the drop to replace the evaporated liquid on the surface. The second phase occurs when there is no longer enough moisture to maintain a saturated state on the surface of the droplets, thus, a dried shell is formed around the surface of the droplet; 5) separation, the final phase, where cyclones, bag filters, and/or electrostatic precipitators are used (Bahr et al., 2020).

At present, frozen storage at −80°C is generally the best complex storage method (Zhang et al., 2020). Nevertheless, for the right long-term storage and stability of EXs (depending on the use of different sources and different experimental techniques), a well-chosen temperature has a significant influence. Some studies have found that, in comparison with freshly extracted EXs, storage at −80°C for 4 days can change the morphology of EXs (Maroto et al., 2017) and that the biological activity of EXs can be decreased during storage at −80°C for a period longer than 28 days (Lorincz et al., 2014). Kalra H. et al. (2013) observed that exosomal recovery rate is not dependent on the viscosity of plasma or the instability of EXs after freezing at −80°C (Kalra et al., 2013). However, at the same time, some other studies have shown that milk-derived EXs are stored at −80°C for 4 weeks without any change in their physical properties (Agrawal et al., 2017). Therefore, it is necessary to study the storage stability of EXs from multiple perspectives (e.g., in terms of source, application technology or future research direction), especially for long-term storage.

## Influence of EXs Isolation Method for Downstream Application

EXs can serve as indicators of normal biological processes, pathological development, or pharmacological response to a therapeutic intervention. The good biomarkers are sensitive, specific, noninvasive, and efficiently assayed using a fast and relatively inexpensive method (Alvarez et al., 2012). The isolation method strongly determines which subpopulations of EXs are captured, which in turn determines how suitable resulting samples are for various downstream applications (Buschmann et al., 2018).The isolation methods of EXs fundamentally influence EV composition (proteins, DNA, RNA or other studied compounds), size, concentration, purity and functionality (Abramowicz et al., 2016; Zhao et al., 2016). Moreover, the source of EXs can influence the choice of isolation method.

There is also a fundamental issue that is not satisfactorily addressed, i.e., the technical standardization of exosome isolation (Witwer et al., 2013). Unfortunately, no standard methods are available for exosome extraction. A few groups have conducted comparative studies on different methodologies (Witwer et al., 2013; Rekker et al., 2014; Greening et al., 2015; Royo et al., 2016). The most often used methods include ultracentrifugation, density gradient centrifugation, chromatography, filtration, polymer-based precipitation and immunoaffinity. Based on the high sensitivity of current molecular techniques, even minor components of EXs can be detected and identified. Thus, the co-isolation of contaminating non-exosomal material can generate a significant artifact (Taylor and Shah, 2015).

### Limitations of EXs Isolation Methods

Each of the most often used EXs isolation methods carry some limitations, in view of the EXs further characterization or application. Ultracentrifugation (UC) can reduce the amount of non-EV particles co-isolated with the EVs, however, it also results in reduced particle yield, due to lost and damaged EVs during high speed centrifugation. Polymer-based precipitation uses volume-excluding polymers to reduce the solubility of EVs and similarly sized proteins and particles, which are subsequently isolated using low speed centrifugation. However, polymer-based approaches require the use of protein removal kits to reduce the amount of co-precipitated proteins. Size-exclusion chromatography (SEC), using e.g., the IZON qEV columns, allows EVs larger than 70 nm to be separated from smaller particles and proteins that take longer to pass through the column. However this method does not completely separate EVs from non-EV material, and the presence of EVs in multiple fractions leads to dilute samples that often need to be pooled and requires an additional concentration step. Density gradient centrifugation uses a density gradient medium such as iodixanol and centrifugal force to separate and purify EVs based on their buoyant density. A second ultracentrifugation step is then required to isolate EVs from a fixed density range, which can result in the loss and damage of some EVs while co-isolating non-EV particles of a similar density.

Faster and more efficient methods to isolate EXs including ultrafiltration (UF) (Cheruvanky et al., 2007), also carry their own set of limitations. For example, highly abundant proteins present in urine tend to be retained by UF, and subsequently interfere with the identification of exosomal proteins (Rood et al., 2010).

### EXs Isolation Method in View of Downstream Proteomic Analysis

The most widely used method for the isolation of EXs from biological fluids, is based on a two-step differential UC (Alvarez et al., 2012). Although this method efficiently isolates EXs, it is time-consuming and requires expensive equipment. The UC method provided the lowest exosome recovery rate, but a higher protein purity of EXs obtained by UC than e.g., with ExoQuick and TEI. Therefore, UC is more suitable for proteomic research. Centrifuging at the high speeds used for EXs isolation could also possibly lead to fusion of the particles with contaminants and other proteins, affecting the physical properties of the EXs and the sensitivity of proteomic analysis (van der Pol et al., 2012; Linares et al., 2015)). It can also cause loss of EXs from the sample, leading to lower and more variable exosome yield (Alvarez et al., 2012).

Three different techniques: UC and two commercial kits, exoEasy (EE; membrane affinity spin column method) and ExoQuick (EQ; polymer-based precipitation) for EXs isolation of patients with breast carcinoma, and further cytokine content analysis were compared (Jung et al., 2020). The dramatic enrichment of CD63 in EXs isolated by UC, but not from EE and EQ, was observed. The high protein content and the low particle-protein ratio together with the low levels of exosome markers isolated from the EQ and EE methods suggest co-isolation of contaminating serum proteins. The cytokine expressions per exosomal particle were the highest in the UC method, which seems to be the most appropriate method when isolating EXs from blood for cytokine profiling. The UC method, however, needs to be tightly controlled because the yield varies with several parameters such as rotor type, sample viscosity, and centrifugation time (Linares et al., 2015).

Kalra H et al. optimized methods to isolate EXs from blood plasma, based on three exosome isolation techniques; differential centrifugation coupled with UC, epithelial cell adhesion molecule immunoaffinity pull-down, and iodixanol density gradient separation. The iodixanol density gradient method was superior in isolating pure exosomal populations, devoid of highly abundant plasma proteins (Kalra et al., 2013). (Gemoll et al., 2020) demonstrated that EV protein expression is highly dependent on the isolation approach. On the proteomic level, UC and ExoQuick seem to be two complementary approaches allowing the detection of different proteoforms with different abundance and purity levels (Gemoll et al., 2020). Thereby, engagement of highly standardized operating procedure for EV isolation, handling and analyzing in combination with an increased transparency for data reporting are needed for implementation of non-invasive EV-based biopsies for cancer diagnostic and prognostic.

### EXs Isolation Method in View of Downstream Transcriptomic Analysis

Similar exosomal small RNA profiles from iodixanol gradient and UC exosome isolation methods, suggesting that the presence of remaining cell debris does not influence downstream transcriptomic analysis, in particular, for the detection of small RNA biomarkers, was observed by Queck et al. (Quek et al., 2017). The commercial kits for EXs isolation are robust, fast, use little sample, and hence serve as ideal choices for the identification of exosomal miRNA disease biomarkers. Compared to UC as the most commonly used method to isolate EXs from plasma/serum, higher miRNA yield in serum EXs was detected by some commercial precipitation kits compared with UC (Keller et al., 2011).

Alvarez et al. (2012) also showed that the exosome isolation method based on the commercial precipitation reagent ExoQuick-TC, yields the highest quantity and quality of exosomal miRNA and mRNA from urine (Alvarez et al., 2012). Helva et al. described a disproportionate relationship between the concentration of particles and the concentration of miRNAs after EXs isolation using UC and exosome isolation kits (miRCURY, ExoQuick, and Invitrogen Total Exosome Isolation Reagent). This is probably due to the heterogeneity of exRNA distributions across the exosome populations and the potential contamination of non-exosome particles including high-density lipoproteins and RNA binding proteins aggregates, which was reported previously (Vickers et al., 2011; Mikaelian et al., 2012; Chevillet et al., 2014). However, the majority of these kits isolate or precipitate EXs and inevitably suffer from the co-isolation of other EVs and protein complexes (Helwa et al., 2017).

There are outstanding publications comparing different strategies for isolating EVs from human serum for RNA analyses that relied on RT-qPCR to profile vesicular miRNAs, (Rekker et al., 2014; Andreu et al., 2016; Crossland et al., 2016). Buschman et al. comprehensively analyzed vesicular microRNAs (miRNAs) in patient biofluids for biomarker studies (Buschmann et al., 2018). They compared five different methods of EVs isolation in combination with two RNA extraction methods regarding their suitability for biomarker discovery. Their findings reveal that isolation by precipitation and membrane affinity was highly suitable for miRNA-based biomarker discovery; whilst methods based on SEC failed to separate patients from healthy volunteers. Isolated vesicles differed in size, quantity, purity and composition, indicating that each method captured distinctive populations of EVs, as well as additional contaminants (Rekker et al., 2014).

Comparative data on methods for isolating EVs from patient biofluids are scarce, despite clear interest in utilizing EVs and their miRNA cargo for biomarker studies. Although a strong correlation of exosomal miRNA profiles was observed between the UC and ExoQuick precipitation method from blood serum of healthy individuals followed by determination of the expression profile of 375 miRNAs by real time PCR using Exiqon miRCURY LNA™ microRNA Human panel I assays, distinct clusters of miRNA levels between the used methods were identified. The detected levels of two miRNAs, miR-92a and miR-486-5p, were significantly. influenced by the exosome isolation method used (Rekker et al., 2014).

### Conclusion Remarks to Influence of Isolation Methods for Downstream EX Content Analysis

The choice of EV isolation method used should depend also on the amount of starting material together with the downstream application, and will be influenced by the need to remove all, or only distinct groups, of non-EV serum components. A recent survey conducted by Gardiner et al. revealed that although UC remains the most commonly used isolation method, other approaches have gained preference when starting volume is limited (Gardiner et al., 2016). It was shown that both qEV and exoRNeasy recover more particles from sepsis sera (Buschmann et al., 2018). Studies relating to small volumes of serum or plasma have either compared different commercial EV isolation kits (Macías et al., 2019) or compared commercial EV isolation kits to size exclusion columns or UC (Andreu et al., 2016; Takov et al., 2019). Density gradients have not been compared and are normally used for large volume EV isolations, such as cell culture conditioned media (Lobb et al., 2015).

We can conclude that most evidence clearly indicates a significant failure to recover exosomal protein and RNA following UC and density gradient centrifugation (Taylor et al., 2011). SEC appears to be a better alternative for exosome isolation, since there is no loss of EXs or damage to the vesicle structure. However, like the centrifugation approaches, total EXs are isolated by this approach. Most studies support the finding that immunoaffinity capture can isolate both total EXs and pathology-specific EXs, while maintaining vesicle integrity and cargo content (Mathivanan et al., 2010; Tauro et al., 2012). Since all of these isolation approaches have limitations, new technologies are being developed, but not commonly available as field flow fractionation (FFF) (Contado, 2017). While still being developed, FFF is based on laminar flow of particles in a solution, where a mixture of particles is propelled through a channel, perpendicular to the direction of flow, resulting in separation of the particles present in the suspension. This method is unique from other separation methods, since it can separate components over a wide colloidal size range.

## Biomedical Applications of EXs

**FIGURE 2:**
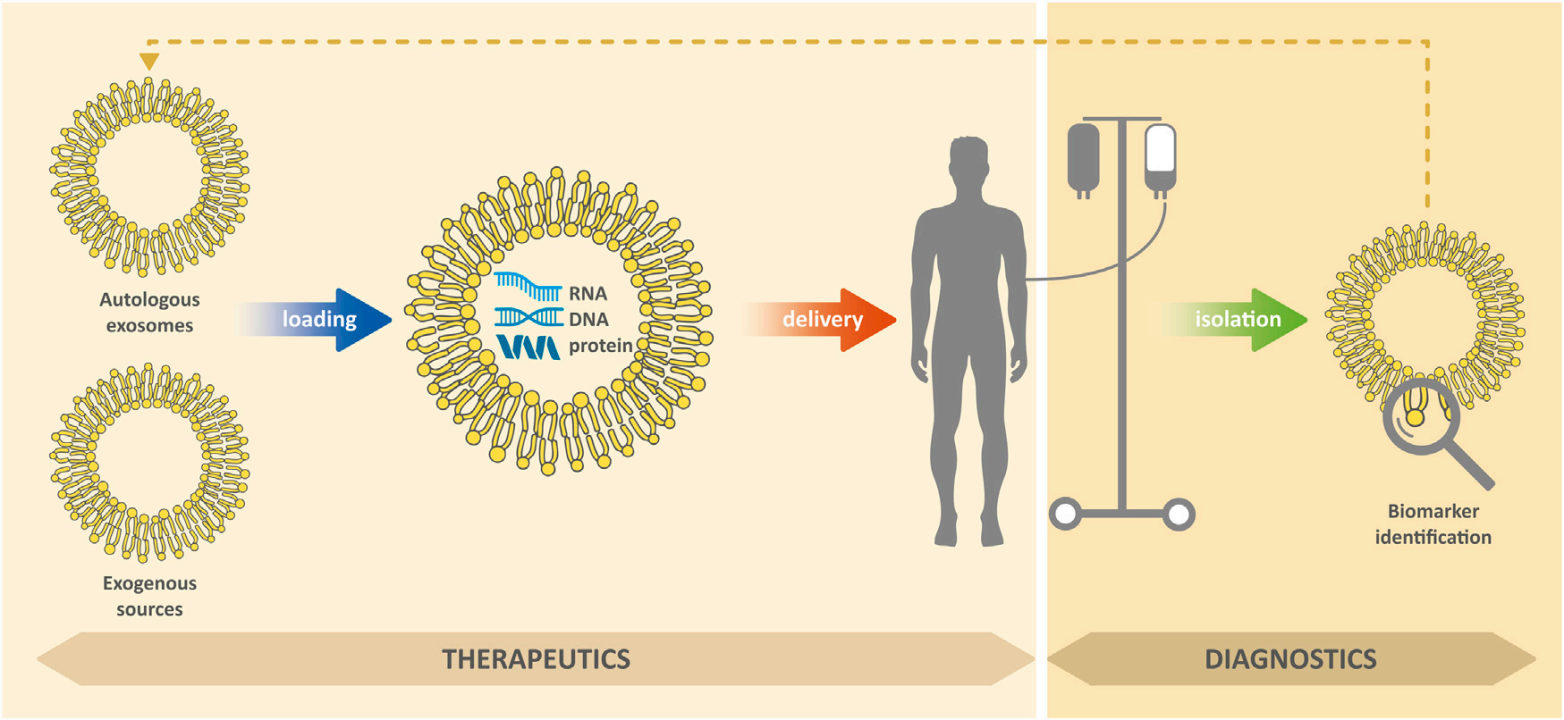
Exosomes for diagnostic or therapeutic purposes.

EXs in body fluids can carry markers of various diseases, including biomolecules, which are otherwise difficult to detect and which (due to lipid membrane) can be protected against degradation (Raimondo et al., 2011; Tu et al., 2011; Lin et al., 2015). In general, successful treatment of diseases usually depends, among other things, on their timely detection. Due to their presence in readily available body fluids (such as blood or urine), they are a valuable biomedical tool for non-invasive diagnostic approaches for cancer, neurodegenerative, cardiovascular and other diseases (Shi et al., 2015; Hirshman et al., 2016; Patel et al., 2019; Deb et al., 2021). In addition, EXs can be used as therapeutic agents to modulate inflammation and promote tissue regeneration, novel modulators for vaccination, or as delivery systems to deliver therapeutic molecules to target cells (Deb et al., 2021; Kim et al., 2021); see Figure 2. However, for medical applications research is necessary, in particular: 1) the ensuring the isolation of pure EXs with a minimum of contaminants; 2) the development of standardization of methods for their isolation; 3) the testing of efficacy and safety of their administration.

### EXs in Diagnostics

The specific nature of EXs can be used, inter alia, for the prediction of cancer. An example is a study of lung adenocarcinoma, in which it has been reported that circulating miRNAs may act as potent biomarkers overall, helping to differentiate patients from healthy controls (Rabinowits et al., 2009). Many miRNAs (e.g., miR-17-5p, miR-10b, miR-550, miR-21) have been isolated from several different EXs obtained from the peripheral blood of pancreatic cancer patients. Subsequently, these miRNAs have been found to be an effective tool for cancer screening (Qiu et al., 2018). It has also been found that the level of miR-373 is highly prominent in the serum of breast cancer patients (Eichelser et al., 2014). Studies of gastric cancer have revealed that an exosomal load-containing long non-coding RNAs (such as INHBA-AS1, CEBPA-AS1, UCA1, AK001058, MIR4435-2HG and LINC00152) is a potent biomarker in blood samples (Ke et al., 2017).

Research into ovarian cancer has shown that EXs containing a tight linker protein claudin-4 are present in the peripheral blood. Several reports indicate that adaptation of the sensitive assay technique for determination of claudin-4 levels can subsequently be used as an effective screening criterion for this type of cancer (Li et al., 2009). Several other studies have revealed other indicators (biomarkers) of various types of cancer, such as for example the presence of excessive amounts of proteoglycan (glypican-1) in EXs obtained from patients with pancreatic cancer, or the presence of MIF indicating the occurrence of liver metastases (Costa-Silva et al., 2015).

As was mentioned before, in addition to cancer as one of the most common serious diseases today, exosomes can also be used in the diagnosis of various other illnesses, such as various neurological, renal or cardiovascular disorders.

The research of various inflammatory and signaling molecules, proteins and RNAs, which occur in the cerebral EXs may serve as a suitable tool in the detection of a different spectrum of neurological disorders (Shaimardanova et al., 2020). Because EXs can cross the blood-brain barrier (BBB) (Xu et al., 2017); they can be isolated from cerebrospinal fluid (CSF) or even blood plasma for diagnostic use (Stuendl et al., 2016).

One of the biomarkers that are present in EXs (and which are also potential players in the pathogenesis of the disease) is protein. For example, in the case of chronic traumatic encephalopathy, which is very common in athletes, so-called tau proteins are found in increased values in plasma EXs (Stern et al., 2016). Another example is the reduction in the level of functional synaptic proteins in Alzheimer’s disease as well as frontotemporal dementia. Decreased functional levels of these proteins are considered a potential biomarker, because in the early stages of the disease the synaptic dysfunction is noticed. The value of proteins in astrocyte-derived EXs is an indicator of patients’ cognitive functions and also prognostic potential (Goetzl et al., 2016).

Extracellular RNAs, which are also found in neuronal cell-derived EXs, are currently considered to be the most potential markers of disease (Rao et al., 2013). Any changes in RNA levels are considered anomalies and indicate a disease state, because circular RNA, miRNA, or even long non-coding RNAs are key to maintaining human cellular homeostasis. Specific examples are circulating fragments of U2 small nuclear RNAs, which are considered a useful biomarker in primary CNS lymphoma (Baraniskin et al., 2016) or levels of long non-coding RNAs RP11462G22.1 in Alzheimer’s and Parkinson’s disease (Gui et al., 2015; Song et al., 2021). Some studies in this area have shown the diagnostic and prognostic role of miRNAs also in trauma, where miR-212 levels decrease and, on the contrary, miR-7a, miR-21, miR-7b and miR-146 expression levels increase (Harrison et al., 2016; Meissner et al., 2016).

Cardiovascular disease is one of the main causes of mortality worldwide. This disease causes almost 20 million deaths a year. In this case, EXs play an important role in regulating the development of disease through the transport and exchange of signaling molecules (Sahoo and Losordo, 2014; Barile et al., 2017). Some studies have shown that EXs regulate processes such as apoptosis, angiogenesis, fibrosis and inflammation in cardiac tissues (Guo et al., 2020). In the cardiovascular system, the number and content of EXs vary depending on physiological conditions (Zamani et al., 2019). Injured cardiomyocytes have been found to release EXs that contain certain miRNAs (e.g., miRNA-1 and miRNA-133a) specific for the heart. Thus, myocardial infarction and any other acute coronary syndrome can be easily identified by controlling the expression of these miRNAs (Kuwabara et al., 2011).

Research has also shown that these miRNAs are upregulated in myocardial infarction and peri infarction in patients with angina pectoris (Cheng et al., 2012). A study by Matsumat et al. revealed that EXs also overexpress miRNA-34a, miRNA-192 and miRNA-194 in heart failure (Matsumoto et al., 2013). At the same time, it has been found that also miRNA-92 is upregulated in such affected patients (Goren et al., 2012). Exosomal proteins may indicate disease states. Dejong et al. described that EXs obtained from hypoxic cardiac cells contain collagen, fibronectin, and lysyl oxidase (de Jong et al., 2012). It has also been found that changes in exosomal proteomes occur after heart transplantation (Kennel et al., 2018).

All types of renal cells, such as glomerular epithelial cells, podocytes, proximal/distal epithelial cells and collecting duct cells (Miranda et al., 2010), secrete EXs. These can also be generated in very small quantities from the systemic circulation (Cheng et al., 2012). However, it can be assumed that most urinary EXs originate from the kidney. EXs secreted by the kidneys contain several biomolecules that could indicate possible abnormalities in the kidneys (Prabu et al., 2019), such as in the case of acute renal injury, where there is (with reference to control, healthy subjects) elevated GPRC5B levels, making this protein a possible diagnostic marker for this type of disease (Kwon et al., 2014). In addition to GPRC5B, ATF3 (activating transcription factor-3) and Fetuin A (Zhou et al., 2006) are also biomarkers indicating acute renal injury.

Other possible and effective biomarkers marking glomerular as well as tubular damage further include, for example, neutrophil gelatinase-associated lipocalin (NGAL) and Wilms Tumor 1 homolog (WT1). The biomarker WT1 was obtained from EXs isolated from the urine of patients suffering from focal segmental glomerulosclerosis (Zhou et al., 2013) and also from a maximum of diabetic patients (Kalani et al., 2013). An above standard level incidence of NGAL was then reported in patients with delayed graft function after kidney transplantation (Alvarez et al., 2013). In contrast, the absence of Prominin-1 (CD133) was indicated as a biomarker in the end-stage renal disease (Dimuccio et al., 2014).

Formerly, renal mRNA has been widely used as a diagnostic and prognostic tool for renal disorders. Current research is also focused on the possible use of miRNA obtained by non-invasive isolation from urinary EXs. One study reported that in a patient with diabetic nephropathy, levels of exosomal miR-130a and miR-145 were found to be elevated from normal and levels of miR-424 and, contrary, miR-155, were decreased (Barutta et al., 2013). Recently, several biomarkers for chronic kidney disease and renal fibrosis have also been characterized. Levels of exosomal miR-29a, miR-29c, miR-200b and miR-200c have been shown to be observed in patients with moderate disease to advanced fibrosis (Lv et al., 2013).

### EXs in Therapy

EXs have several characteristics suitable for drug delivery. These include nano-size, non-toxicity, biocompatibility, low immunogenicity as well as targeting ability and organotropis (Lim and Kim, 2019). The similarities between EXs and liposomes in size and ability to carry both hydrophilic and lipophilic molecules is obvious. However, EXs’ asymmetric lipid distribution and specific protein composition of their membranes justify their organotropism and ability to target (Antimisiaris et al., 2018). This is supported by evidence that cancer-derived EXs preferentially fuse with their parent cells (Qiao et al., 2020). However, the clinical use of EXs as drug carriers is fraught with several technical problems, such as: 1) relatively low production yields; 2) considerable structural heterogeneity; 3) difficulties in drug loading 4) the development of standard, scalable and cost-effective procedures for their isolation and purification (Lu and Huang, 2020).

To overcome these problems, bioinspired exosome vesicles have been developed as an alternative to naturally derived Exs. Most of the, until now proposed, artificial exosomal mimetic systems, so-called hybrid Exs, are formed by the fusion of exosomal and liposome membranes (Sato et al., 2016) or are obtained by serial extrusion of a mother cell suspension by reducing pore size (Jang et al., 2013).

Exs are currently seen as ideal candidates for the delivery of antitumor agents, miRNAs, siRNAs, and various other biomolecules with therapeutic potential. Possible therapeutic targeting of Exs involves cell targeting, altering the native result of Exs (so that the process of tumorigenesis can be disrupted by interactions with tumor cells), stromal cells, and other immune cells (Guo et al., 2020; Deb et al., 2021).

It has been found that the removal of Exs from the circulatory system appears to be a possible functional treatment option to eliminate the metastatic effect of cancer (Tickner et al., 2014). At the same time, some studies also suggest that inhibition of exosome generation can arrest tumor formation using several different techniques, such as proton pump inhibition, microtubule assembly targeting, or endosomal sorting (Pilzer et al., 2005; Azmi et al., 2013).

Some studies suggest that the use of Exs in the treatment of cancer for the delivery of chemotherapeutic agents appears promising (Yeo et al., 2013). For example, the growth of cancer cells in breast and colon tumors has been successfully inhibited by doxorubicin-containing Exs or by mimetic exosomal nanosvesicles (Jang et al., 2013; Tian et al., 2014; Smyth et al., 2015). Based on the observation that exosomal microRNAs act effectively on the target mRNA and thus suppress gene expression in recipient cells, cancer research has also focused on delivering a specific miRNA or a small useful amount of interfering RNA (siRNA). Preclinical testing for the delivery of therapeutic miRNAs or siRNAs via Exs has focused, for example, on antitumor therapy in rodents with breast cancer (Ohno et al., 2013), glioma (Katakowski et al., 2013) or pancreatic cancer (Kamerkar et al., 2017; Mendt et al., 2018).

Further studies also indicate that Exs can be a successful alternative for the delivery of antigens which are derived from a tumor to elicit an immune response (El Andaloussi et al., 2013). Some studies based on this finding are already in the clinical trials phase (Escudier et al., 2005; Morse et al., 2005; Dai et al., 2008). It has been found that Exs play a role in exosome-mediated cell communication in cancer and that Exs derived from cancer stem cells are involved in their differentiation (Koch et al., 2014; Lin et al., 2015; Parimon et al., 2018). It has been observed that Exs participate in conferring resistance to cancer cells as well (Qu et al., 2016). All of the above studies (and many others) suggest that tumor cell-derived Exs indeed play a key role in both cancer cell proliferation and their possible elimination. Finally, they may also play a crucial role in the potential resistance to some drugs.

There are several causes that can lead to CNS tissue damage. These include injuries, hereditary diseases, infections, ischemia and many others. The fact that the BBB is only very selectively permeable is a major limitation in the treatment of CNS diseases (Timbie et al., 2015). Therefore, recent research has focused on finding various new techniques for successful BBB penetration, an example being the use of nanoparticles (Zhang and Yang, 2018).

In addition to being a very potent biomarker, EXs are potential candidates for drug delivery. EXs are highly desirable as drug carriers, especially because of their nanoscale dimensions, ability to cross the BBB, and protection against degradation - they can retain their original state (Kourembanas, 2015). However, in addition to their advantageous properties, EXs have a disadvantage as well, they were shown to assist the progression of some diseases by spreading pathogenic agents into healthy cells (Bakhshandeh et al., 2016). Despite this possible disadvantage, the potential use of EXs has been identified in several neurodegenerative diseases such as Parkinson’s disease (Zhao et al., 2014), Alzheimer’s disease (Alvarez-Erviti et al., 2011; Stuendl et al., 2016), Huntington’s disease (Didiot et al., 2016), epilepsy (Longa et al., 2017), multiple sclerosis and amyotrophic lateral sclerosis (Rajan et al., 2016).

A number of studies have been presented on the role of EXs in myocardial infarction. Some studies revealed that customized CD34 ^+^ stem cells can release EXs containing pro-angiogenic factors and a sound hedgehog (Shh) (MacKie et al., 2012). These specifically treated CD34 + cells, when injected into a mice model of acute myocardial infarction, showed a protective function against ventricular dilatation (MacKie et al., 2012). Another study showed that EXs derived from mesenchymal stem cells can increase the division of M1 macrophages into M2 macrophages, resulting in a significant enhancement of the phenomenon of inflammation in cardiac tissues and thus a reduction in infarct size.

Another study reported that exosomal miR-19a derived from the aforementioned cell type has anti-apoptotic effects caused by activation of the AKT protein, targeting SOX6, and blockade of the JNK3/caspase-3 pathway (Lu and Huang, 2020). EXs from cardiosphere-derived cells have been described as possible therapeutic agents in the treatment of cardiovascular disease. In the case of post myocardial infarction, these EXs were found to be able to enhance cardiac fibrosis. At the same time, it has been observed that, in cardiomyocyte apoptosis, the process of endothelial cell tube generation is impaired (Tseliou et al., 2015; Namazi et al., 2018).

Research revealed that another promising nanotechnology tool that can be used for treatment of cardiovascular diseases is cardiac progenitor cell (CPC) derived EXs (Guo et al., 2020). Studies in vivo models of myocardial infarction have shown that CPC-derived EXs lead to an increase in miR-210, resulting in a decrease in ephrin A3 and PTP1b, causing a decrease in apoptosis in cardiomyocytes (Barile et al., 2014). These EXs are also able to activate PAPP-A, leading to the release of IGF-1 and ultimately increasing intracellular Akt and ERK1/2 (Barile et al., 2014).

In addition to the above-mentioned uses as biomarkers, EXs also offer a number of therapeutic possibilities in kidney diseases. Examples include their use in modulating kidney transplant rejection, correcting several metabolic deficiencies, or promoting renal regeneration (Deb et al., 2021). A group of researchers, led by Grange, demonstrated that labeled MSC-Exos administered intravenously was able to target the injured kidney (Nassar et al., 2016). In another study, it was observed that, in patients with stage 3 or 4 chronic kidney disease, MSC-EXs were able to extemporize the glomerular filtration rate and also reduce albumin excretion (Dorronsoro and Robbins, 2013).

Other research has shown that repeated administration of EXs can prevent the development of chronic tubular injury. EXs isolated from bone marrow mesenchymal cells and umbilical cord mesenchymal cells have a protective effect against cisplatin-induced nephrotoxicity in both *in vivo* and *in vitro* models. The presumed reason is probably the fact that the human umbilical cord MSCs-Exo can successfully reduce the level of Bax (a protein similar to 4 bcl-2) and, conversely, increase Bcl-2 (B-cell lymphoma 2) to prevent apoptosis and activate the Erk1/2 pathway, leading to increased proliferation after cisplatin-induced renal injury (Dorronsoro and Robbins, 2013).

Diabetes is one of the main causes of chronic kidney disease, especially in developed and Western countries. Around 40% of patients with this disease develop diabetic neuropathy as the main complication (Prabu et al., 2019). Jiang et al. published the results of a 1-week study in intravenous EXs and studied their effect on renal damage and streptozotocin - induced angiogenesis in a Sprague-Dawley rat model. These results showed a reduction in urine volume and microalbuminuria as well as suppression of apoptosis of tubocytes and tubular epithelial cells induced by high glucose (Jiang et al., 2016). Some of the other remarkable work done with the EXs in the field of renal treatment include, for example, studies of Jia et al. (Jia et al., 2018), Sanches et al. (Sanchez et al., 2014) or Cantaluppi et al. (Cantaluppi et al., 2012).

### Role of EXs in Senescence and Aging

Senescence is the cellular part of tissue aging due to irreversible growth arrest and other physiological changes that occur in the morphology, behavior and function of cells. Aging is also a well-known risk factor in the development of many human diseases, such as neurodegenerative disorders, cardiovascular disease, or cancer, which are common causes of death or invalidity, especially in the elderly population (Xu and Tahara, 2013). miRNAs, that regulate gene expression, play an important role in many biological processes. In terms of aging, some miRNAs were proven to be key regulators during cell aging (Weilner et al., 2013). Many studies have already shown that EXs contain miRNAs and play an important role in cell to cell communication and information transfer (Simons and Raposo, 2009; Camussi et al., 2010; Mathivanan et al., 2010; Bang and Thum, 2012). EXs with miRNAs were found to participate in the senescence of a complex cell network and contribute to aging. There is clear evidence that aging increases the number of circulating EXs in tissues. Especially from aging cells, the secretion of immunosuppressive EXs is strongly increased.

There are studies that have shown that EXs from aging cells are relatively significantly involved in the expansion of aging into neighboring cells (Alibhai et al., 2020; Salminen et al., 2020). Very interestingly, age-related EXs also contain, among other things, immunosuppressive costs that increase immunosuppression in recipient immune cells, i.e., tissue resident and accepted immune cells, e.g., M2 macrophages, myeloid-deriv ed suppressor cells (MDSC) or regulatory T cells (Salminen et al., 2020). It seems that senescent cells secrete immunosuppressive EXs in an attempt to escape the immune surveillance, and their accumulation within tissues consequently augments the aging process (Xu and Tahara, 2013; Salminen et al., 2020).

### EXs and COVID-19 Virus Infection

Since December 2019, the SARS-CoV-2 (COVID-19) pandemic has become a serious public health problem worldwide. Although a number of vaccines already exist today (e.g., Pfizer and BioNTech, Moderna, Astra-Zeneca, Johnson and Johnson), finding a safe and effective prognostic, diagnostic and therapeutic tool for patients, especially those with severe COVID-19 infection, is still an actual theme. Knowledge of the host response to the new type of coronavirus SARS-CoV-2 remains limited, which prevents a detailed understanding of the pathogenesis of the disease and, consequently, the development of effective therapeutic strategies (Hassanpour et al., 2020) (Hassanpour et al., 2020). It is common for host cells to release EXs (and other extracellular vesicles) that carry viral as well as host components that can modulate the immune response during viral infection (Tan et al., 2016; Kurywchak et al., 2018; Li et al., 2019). The same seems to be the case with this disease.

In one research focused on the role of EXs in Covidem-19 infection, the scientific team asked several basic questions for this statement: 1) does COVID-19 virus use EXs and other extracellular vesicles for infection? and 2) can extracellular vesicles be targeted for the treatment of COVID-19 virus infection? The results of this study revealed that EXs from COVID-19 infected cells may contribute to spread and infection. EXs contain receptors for COVID-19 virus such as CD9 and ACE2, which may be involved in promoting COVID-19 virus infection. At the same time, the possibility of preclinical and clinical application of EXs for the treatment of COVID-19 infection was outlined, which would include stem cell-derived exosome therapy, exosome-based drug delivery, possible inhibition of exosome biogenesis and absorption, and, last but not least, a new exosome-based vaccine (Hassanpour et al., 2020).

A study by Barberis et al. investigated how SARS-CoV-2 infection affects exosome content, the involvement of EXs in disease progression, and the possible use of plasma EXs as biomarkers in determining disease severity. The results of proteomic analysis of EXs obtained from patients with this type of virus identified several molecules that participate in immune responses, inflammation, and activation of coagulation and complement pathways, which are likely to be major mechanisms leading to tissue damage and subsequent multi-organ dysfunction. Moreover, several potential biomarkers, such as fibrinogen, fibronectin, the C1r subcomponent of complement and the P-component of serum amyloid, have been shown to correlate with established disease severity. In addition, the presence of SARS-CoV-2 RNA in the exosomal load was also detected in this study, supporting the hypothesis that the virus may use the endocytosis pathway to spread infection (Barberis et al., 2021).

Another study addressed the issue of relapse of COVID-19 infection. The authors hypothesized that one of the possible mechanisms of relapse of this infection may be a cellular transport pathway associated with the release of EXs containing SARS-CoV-2 and other extracellular vesicles. The results suggest that this so-called “Trojan horse” strategy provides a possible explanation for viral RNA recurrence, even in recovered patients with COVID-19 7–14 days after discharge. It appears that viral material could be hidden in EXs or other extracellular vesicles during this time and then spread again (Elrashdy et al., 2020) (Elrashdy et al., 2020).

Several other current studies were focused on the use of EXs (e.g., from mesenchymal cells) as nanoplatforms for therapeutics and drug delivery in the fight against COVID-19 (Alzahrani et al., 2020; Sengupta et al., 2020; Pinky et al., 2021), exosome-mediated mRNA delivery for SARS-CoV-2 vaccination (Tsai et al., 2020) or their potential role on cytokine storm and treatment (Kadriyan et al., 2020).

## Conclusion

EXs are now well known as important mediators of cell to cell communication, as biomarkers, and as crucial players in both physiological and pathological processes, due to their secretion from various types of cells, and detection in almost all kinds of body fluids. The exosome capacity to envelope a wide range of content, including lipids, RNAs, proteins or therapeutics, to signal specific recipient cells or tissues, makes them a promising diagnostic and therapeutic nanosize tool.

In addition to conventional (usually mammal) sources of EXs, there is a wide range of “nonconventional” (non-mammalian) sources of EXs, which are at present intensively studied, mainly for their inter-species and inter-kingdom communication, which can have important environmental, ecological or medicinal impact.

Furthermore, the isolation and purification methods of EXs is still a “hot topic”, which needs to be standardized for the feasible clinical application of EXs, or given a clearer overview of the advantages and limitations of individual isolation and purification methods, as well as storage conditions, for further downstream EXs characterization or application.

Undoubtedly, EXs represent a promising tool in the field of nanomedicine in diagnostics, therapy, and recently, with more emphasis on senescence and aging, in issues related to Covid-19.

## Supplementary Material

Supplementary Table S1 Overview of main Exosomes isolation methods.
