# Supplementary material for: Conventional and Nonconventional Sources of Exosomes–Isolation Methods and Influence on Their Downstream Biomedical Application
Source: Front Mol Biosci. 2022 May 2;9:846650. doi: 10.3389/fmolb.2022.846650 (PMC9110031; doi:10.3389/fmolb.2022.846650)
Supplement: Supplementary file 1 [file Table1.pdf]

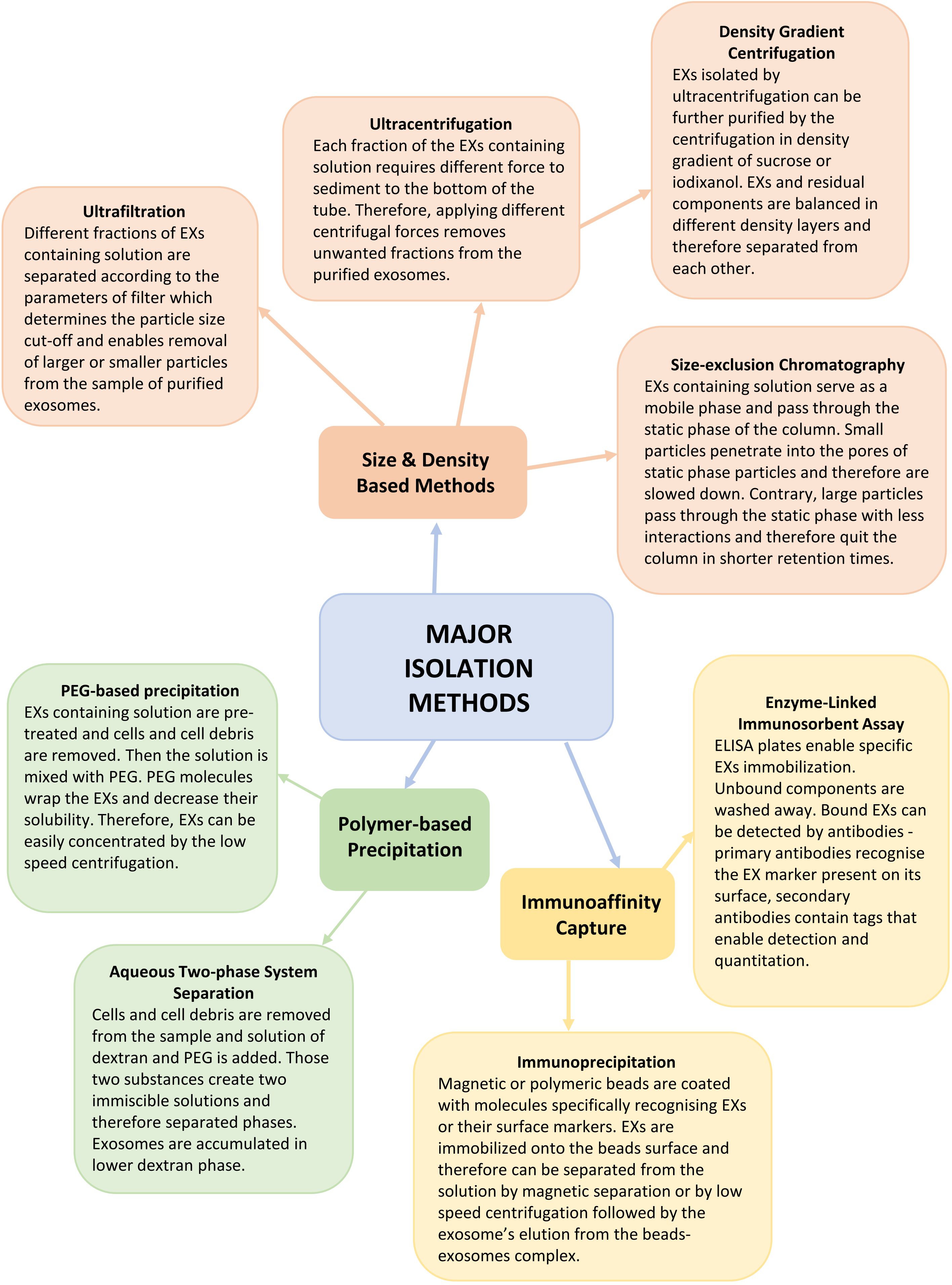

MAJOR ISOLATION METHODS
Size & Density Based Methods
Ultrafiltration
Different fractions of EXs containing solution are separated according to the parameters of filter which determines the particle size cut-off and enables removal of larger or smaller particles from the sample of purified exosomes.
Ultracentrifugation
Each fraction of the EXs containing solution requires different force to sediment to the bottom of the tube. Therefore, applying different centrifugal forces removes unwanted fractions from the purified exosomes.
Density Gradient Centrifugation
EXs isolated by ultracentrifugation can be further purified by the centrifugation in density gradient of sucrose or iodixanol. EXs and residual components are balanced in different density layers and therefore separated from each other.
Size-exclusion Chromatography
EXs containing solution serve as a mobile phase and pass through the static phase of the column. Small particles penetrate into the pores of static phase particles and therefore are slowed down. Contrary, large particles pass through the static phase with less interactions and therefore quit the column in shorter retention times.
Polymer-based Precipitation
PEG-based precipitation
EXs containing solution are pre-treated and cells and cell debris are removed. Then the solution is mixed with PEG. PEG molecules wrap the EXs and decrease their solubility. Therefore, EXs can be easily concentrated by the low speed centrifugation.
Aqueous Two-phase System Separation
Cells and cell debris are removed from the sample and solution of dextran and PEG is added. Those two substances create two immiscible solutions and therefore separated phases. Exosomes are accumulated in lower dextran phase.
Immunoaffinity Capture
Enzyme-Linked Immunosorbent Assay
ELISA plates enable specific EXs immobilization. Unbound components are washed away. Bound EXs can be detected by antibodies - primary antibodies recognise the EX marker present on its surface, secondary antibodies contain tags that enable detection and quantitation.
Immunoprecipitation
Magnetic or polymeric beads are coated with molecules specifically recognising EXs or their surface markers. EXs are immobilized onto the beads surface and therefore can be separated from the solution by magnetic separation or by low speed centrifugation followed by the exosome's elution from the beads-exosomes complex.
